# Chronic type adult T-cell leukemia-lymphoma after autolougous stem cell transplantation for ALK-negative anaplastic large cell lymphoma

**DOI:** 10.1186/1742-4690-12-S1-P25

**Published:** 2015-08-28

**Authors:** Atae Utsunomiya, Masahito Tokunaga, Nobuaki Nakano, Ayumu Kubota, Shogo Takeuchi, Yoshifusa Takatsuka, Noriaki Yoshida, Masao Seto

**Affiliations:** 1 Department of Hematology, Imamura Bun-in Hospital, Kagoshima, Japan; 2 Department of Pathology, Kurume University School of Medicine, Kurume, Japan

## 

An adult male patient was admitted to our hospital with systemic lymphadenopathy. He was diagnosed as having ALK-negative anaplastic large cell lymphoma (ALCL) by lymph node biopsy. Immunohistochemical staining revealed positive reaction for CD4, CD30, CD25, TIA-1, and Granzyme B, but negative for ALK and EBER. Although anti-HTLV-1 antibody in his serum was positive, monoclonal integration of HTLV-1 provirus DNA was not detected in his lymph node by Southern blot analysis. He was treated by combination chemotherapy, and complete remission was obtained by 8 courses of CHOP therapy. Subsequently he received autologous stem cell transplantation (auto-SCT). Fifteen months after auto-SCT, abnormal lymphocytes in the peripheral blood gradually increased. Southern blot analysis of abnormal lymphocytes revealed monoclonal integration of HTLV-1 provirus DNA and monoclonal rearrangement of T-cell receptor b. The patient was therefore diagnosed as chronic type of adult T-cell leukemia-lymphoma (ATL), and immediately progressed to acute type with central nervous system involvement. He died of tumor progression regardless of intensive chemotherapy. We analyzed genomic alterations of the ALCL cells and the chronic type ATL cells using high-resolution array comparative hybridization. These results revealed that the genomic alteration pattern of ALCL was different from those of the chronic type. Chronic type ATL cells had the genetic alterations such as 1q gain and CD58 loss that were frequently observed in acute type ATL than the chronic type, suggesting that these genetic events might contribute to the transformation of this case. TCR rearrangement analyses using PCR were performed for the tumor cells of ALCL and ATL to evaluate the origin of these tumor cells. Results showed that tumor cell clone of the ATL might have already proliferated in ALCL lymph node. This finding suggests that lymph nodes can serve as a niche for ATL development.

